# Effects of Dietary l-Arginine on Nitric Oxide Bioavailability in Obese Normotensive and Obese Hypertensive Subjects

**DOI:** 10.3390/nu8060364

**Published:** 2016-06-14

**Authors:** Beverly Giam, Sanjaya Kuruppu, Geoffrey A. Head, David M. Kaye, Niwanthi W. Rajapakse

**Affiliations:** 1 Baker IDI Heart and Diabetes Institute, VIC 3004 Melbourne, Australia; beverly.giam@bakeridi.edu.au (B.G.); Geoff.Head@bakeridi.edu.au (G.A.H.); david.kaye@bakeridi.edu.au (D.M.K.); 2 Central Clinical School, Monash University, VIC 3004 Melbourne, Australia; 3 Department of Biochemistry and Molecular Biology, Monash University, VIC 3004 Melbourne, Australia; sanjaya.kuruppu@monash.edu; 4 Department of Physiology, Monash University, VIC 3004 Melbourne, Australia

**Keywords:** l-arginine transport, nitric oxide, obesity related hypertension

## Abstract

Obesity related hypertension is a major risk factor for resistant hypertension. We do not completely understand the mechanism(s) underlying the development of obesity related hypertension which hinders the development of novel treatment strategies for this condition. Data from experimental studies and small clinical trials indicate that transport of l-arginine, the substrate for nitric oxide (NO), and subsequent NO production are reduced in obesity induced hypertension. Reduced NO bioavailability can induce hypertension via multiple mechanisms. Mirmiran *et al.* recently analyzed data from a large population study and found that the association between dietary l-arginine and serum nitrate and nitrite was weakened in obese hypertensive subjects compared to obese normotensives. These data suggest that l-arginine dependent NO production is impaired in the former group compared to the latter which may represent a novel mechanism contributing to hypertension in the setting of obesity.

It is increasingly recognized that obesity related hypertension is a strong risk factor for resistant hypertension [1]. Despite this, we still do not completely understand the precise factors that underpin the development of obesity related hypertension leaving obese patients at risk of developing cardiovascular diseases and related complications [1]. Of interest, not all obese patients develop hypertension and why this is so remains a mystery [2]. Whilst genetic factors may play a role in predisposing obese patients to the development of hypertension [3] other factors are likely to contribute as well [1,3]. In this context, Mirmiran *et al.* recently analysed data from a large population study and found that dietary l-arginine can improve serum nitrate and nitrite levels, an index of nitric oxide (NO) bioavailability, in obese subjects [4]. Interestingly, they also found that the association between dietary l-arginine and serum nitrate and nitrite levels was greater in obese normotensives compared to obese hypertensives [4]. These data suggest that l-arginine dependent NO formation is compromised in obese hypertensive subjects when compared to their normotensive counterparts. Findings by Mirmiran *et al.* are in agreement with a growing body of evidence which indicates that impaired l-arginine transport and reduced NO levels are associated with obesity related hypertension [1]. For example, plasma nitrate and nitrite levels were less in obese hypertensive subjects compared to obese normotensive subjects [5]. Furthermore, weight reduction in obese patients was associated with reduced plasma l-arginine levels and improved plasma nitrate and nitrite levels [6] and as expected, weight reduction also led to reduced arterial pressure [6]. This indicates that weight reduction in obese subjects is associated with improved capacity for l-arginine dependent NO formation and normalisation of obesity related hypertension. l-arginine is the sole substrate for NO formation and it has been demonstrated that extracellular l-arginine concentration can affect NO bioavailability [1,7]. This is despite the fact that intracellular l-arginine concentrations far exceed the Michaelis-Menton constant (K_m_) for endothelial NO synthase; a phenomenon commonly referred to as the ‘l-arginine paradox’ [7]. The presence of this paradox in turn renders NO bioavailability to be susceptible to function of l-arginine transporters (Figure 1). Cationic amino acid transporter-1 (CAT1) is the predominant l-arginine transporter expressed in endothelial cells and in the kidney [1,7]. Previously, it was demonstrated that reductions in renal CAT1 expression *per se* can induce hypertension in otherwise normal rats [8]. This indicates that impairments in renal l-arginine transport *per se* can induce hypertension potentially via reducing renal NO bioavailability [8]. Interestingly, we recently found that expression of renal CAT1 is reduced in experimental obesity induced hypertension [5]. Furthermore, augmenting endothelial specific l-arginine transport, including that within the kidney, abolished obesity induced hypertension in mice [5]. These data indicate that reduced l-arginine transport via CAT1 likely plays an important role in the pathogenesis of obesity related hypertension. Furthermore, it has been demonstrated that arginase expression and activity were greater in obese hypertensive rats compared to lean controls [9]. Arginase inhibition normalized blood pressure in these rats suggesting that augmented arginase activity and subsequent reductions in l-arginine levels can contribute to obesity induced hypertension [9]. Augmented arginase activity is also documented in morbidly obese humans [10]. Mirmiran *et al.*’s findings provide direct evidence that dietary l-arginine can improve NO bioavailability in obese normotensive subjects and that this association is weakened in obese hypertensives [4]. Their data suggest that the association between the l-arginine-NO pathway and obesity related hypertension holds true beyond the experimental setting. Further studies are required to determine the mechanism(s) underlying augmented arginase activity, impaired l-arginine transport and reduced NO bioavailability in the setting of obesity. Regardless of the mechanism(s) involved, above data raise an important question as to whether reductions in NO bioavailability are central to the development of obesity related hypertension. Support for this notion comes from findings which indicate that reduced NO levels can induce and maintain hypertension via multiple mechanisms [1]. Further experimental, and in particular, clinical studies are required to assess whether interventional strategies aimed at augmenting l-arginine transport and/or NO bioavailability can halt or reverse obesity related hypertension in man. In particular, development of novel drugs that can specifically target the l-arginine-NO pathway is likely to hold great promise in the treatment of obesity related hypertension.

## Figures and Tables

**Figure 1:**
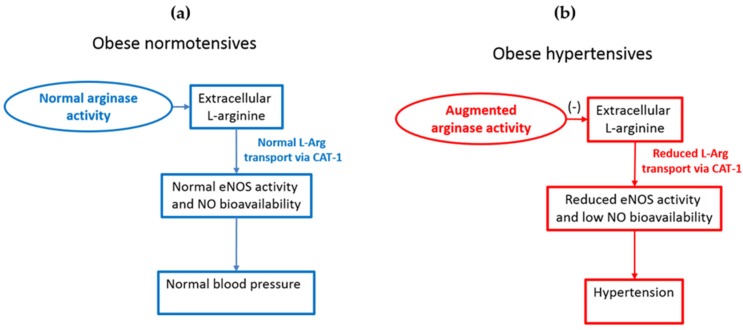
(**a**) Normal arginase activity and normal transport of L-arginine via CAT-1 contribute to the regulation of blood pressure in obese normotensives (**b**) Reduced l-arginine transport via CAT-1 and/or augmented arginase activity can decrease NO bioavailability and thereby increase arterial pressure in obese hypertensives. CAT-1 is the predominant l-arginine transporter expressed in endothelial cells and it is co-localised with eNOS. This allows extracellular l-arginine transported by CAT-1 to be readily available for eNOS dependent NO production. l-Arg, l-arginine; CAT-1, Cationic amino acid transporter-1; eNOS, endothelial nitric oxide synthase; NO, nitric oxide.

## Abbreviations

The following abbreviations are used in this manuscript:

NO	Nitric oxide
CAT-1	Cationic amino acid transporter-1
eNOS	Endothelial nitric oxide synthase
l-Arg	l-arginine
